# Multiomics profiling of the impact of an angiotensin (1–7)-expressing probiotic combined with exercise training in aged male rats

**DOI:** 10.1152/japplphysiol.00508.2022

**Published:** 2023-03-09

**Authors:** Liliana C. Baptista, Emily L. Zumbro, Zachary A. Graham, Abbi R. Hernandez, Taylor Buchanan, Yi Sun, YouFeng Yang, Anisha Banerjee, Amrisha Verma, Qiuhong Li, Christy S. Carter, Thomas W. Buford

**Affiliations:** ^1^Division of Gerontology, Geriatrics and Palliative Care, Department of Medicine, University of Alabama at Birmingham, Birmingham, Alabama, United States; ^2^Research Center for Physical Activity, Health and Leisure, Faculty of Sport, University of Porto, Porto, Portugal; ^3^Laboratory for Integrative and Translational Research in Population Health, University of Porto, Porto, Portugal; ^4^Research Service, Birmingham Veterans Affair Medical Center, Birmingham, Alabama, United States; ^5^Healthspan, Resilience and Performance, Florida Institute for Human and Machine Cognition, Pensacola, Florida, United States; ^6^Department of Cell, Developmental, and Integrative Biology, University of Alabama at Birmingham, Birmingham, Alabama, United States; ^7^Department of Life, Health, and Physical Sciences, Gordon College, Wenham, Massachusetts, United States; ^8^Department of Ophthalmology, College of Medicine, University of Florida, Gainesville, Florida, United States; ^9^Geriatric Research Education and Clinical Center, Birmingham VA Medical Center, Birmingham, Alabama, United States

**Keywords:** angiotensin (1–7), genetically modified probiotic, metabolomics, microbiome, transcriptomics

## Abstract

Angiotensin (1–7) [Ang (1–7)] is an active heptapeptide of the noncanonical arm of the renin-angiotensin system that modulates molecular signaling pathways associated with vascular and cellular inflammation, vasoconstriction, and fibrosis. Preclinical evidence suggests that Ang (1–7) is a promising therapeutic target that may ameliorate physical and cognitive function in late life. However, treatment pharmacodynamics limits its clinical applicability. Therefore, this study explored the underlying mechanisms altered by a genetically modified probiotic (GMP) that expresses Ang (1–7) combined with and without exercise training in an aging male rat model as a potential adjunct strategy to exercise training to counteract the decline of physical and cognitive function. We evaluated cross-tissue (prefrontal cortex, hippocampus, colon, liver, and skeletal muscle) multi-omics responses. After 12 wk of intervention, the 16S mRNA microbiome analysis revealed a main effect of probiotic treatment within- and between groups. The probiotic treatment enhanced α diversity (Inverse Simpson (*F*[2,56] = 4.44; *P* = 0.02); Shannon–Wiener (*F*[2,56] = 4.27; *P* = 0.02)) and β-diversity (*F*[2,56] = 2.66; *P* = 0.01) among rats receiving our GMP. The analysis of microbes’ composition revealed three genera altered by our GMP (*Enterorhabdus*, *Muribaculaceae unclassified*, and *Faecalitalea*). The mRNA multi-tissue data analysis showed that our combined intervention upregulated neuroremodeling pathways on prefrontal cortex (i.e., 140 genes), inflammation gene expression in the liver (i.e., 63 genes), and circadian rhythm signaling on skeletal muscle. Finally, the integrative network analysis detected different communities of tightly (|*r*| > 0.8 and *P* < 0.05) correlated metabolites, *genera*, and genes in these tissues.

**NEW & NOTEWORTHY** This manuscript uses a multiomics approach (i.e., microbiome, metabolomics, and transcriptomics) to explore the underlying mechanisms driven by a genetically modified probiotic (GMP) designed to express angiotensin (1–7) combined with moderate exercise training in an aged male rat model. After 12 wk of intervention, our findings suggest that our GMP enhanced gut microbial diversity while exercise training altered the transcriptional response in relevant neuroremodeling genes, inflammation, and circadian rhythm signaling pathways in an aging animal model.

## Graphical abstract

**Figure:**
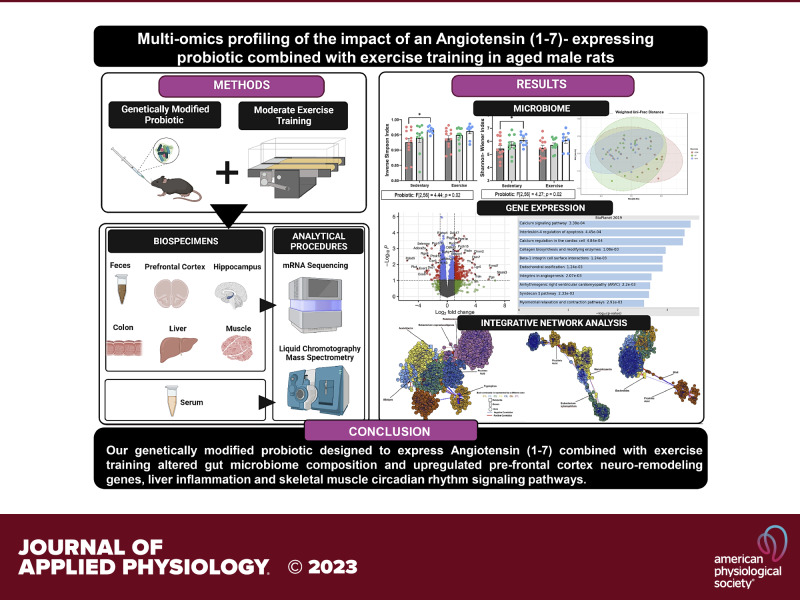

## INTRODUCTION

Physical and cognitive decline are intermediate stages of several age-related diseases that reduce health and lifespan, and ultimately, may lead to physical disability and mortality (1, 2). Multiple factors are involved in the onset of physical and cognitive decline including low-grade chronic inflammation, changes in the cardiovascular, musculoskeletal, and neuronal systems (2, 3) along with alterations on gut microbiota (2–5). Despite the advances in clinical care, to date, treatment options to ameliorate these symptoms and outcomes remain elusive. Exercise training is the only therapy reported to consistently protect from the age-related loss of skeletal muscle mass, strength and quality, and to preserve physical function (6–8). Apart from the functional benefits, exercise training also modulates gut microbiome composition and function in preclinical and clinical studies in adults (9–11) and enhances cognitive function in older adults with cognitive impairment and dementia (12). However, this evidence is inconsistent on exercise effects (10, 11, 13), partly because host genetics, physical fitness, and exercise intensity also influence gut health, physical, and cognitive function (14–16). Therefore, these well-confirmed benefits do not extend to all individuals and there is large interindividual response variability in those that do maintain a regular exercise training regimen (17). In addition, treatment strategies are minimal for those unwilling or unable to engage in regular exercise training. Thus, adjuvant or alternative therapies are needed to optimize the efficacy of exercise training among older adults with physical and cognitive decline.

Over the past two decades, our research group has been focused on modulating components of the renin-angiotensin (RAS) system as an adjuvant/alternative therapeutic avenue to maintain physical and cognitive function in late life when combined with exercise training. Our overarching hypothesis is that by counteracting the activity of the vasoconstrictor RAS arm and/or stimulating the noncanonical pathway of the RAS system may attenuate the age-related decline of physical and cognitive function, when combined with regular exercise training. This hypothesis is supported by multiple lines of evidence of local RAS components within skeletal muscle (18–20), the gastrointestinal tract (21, 22), and throughout the central nervous system (23–26) as well as by the experimental findings from our group (27–29). Recently, due to its growing biological relevance, we have concentrated in the active heptapeptide of the noncanonical arm of the RAS: the angiotensin (1–7) [Ang (1–7)]. Ang (1–7) is cleaved from angiotensin I or II (Ang I/II) by the angiotensin-converting enzyme 2 (ACE2) and operates via a Mas (AT_7_) receptor, counteracting multiple physiological processes led by Ang II. Physiologically, Ang (1–7) modulates molecular and cellular signaling pathways associated with vascular and cellular inflammation, hyperplasia, and fibrosis (30–32), which are cellular catabolic processes involved in the development of physical and cognitive decline.

Interestingly, preclinical evidence shows that therapeutic interventions using Ang (1–7) improve cognition, mitigate Tau-protein deposition, and amyloid-β induced changes in mitochondria bioenergetics and also prevents skeletal muscle atrophy in sarcopenic mice (20, 33–40). Still, despite these benefits on physical and cognitive function in rodent models, the therapeutic potential of Ang (1–7) in humans remains elusive due to its short half-life and low oral bioavailability (41). To overcome these treatment limitations and explore the therapeutic potential of Ang (1–7) on physical and cognitive decline as an adjunct strategy to exercise training, we developed a gut microbiota-targeted therapeutic method using a genetically modified probiotic (GMP) designed to express Ang (1–7). This isolated GMP increased circulating levels of Ang (1–7), while reducing the deleterious levels of Ang II in an aging rodent model (42). We also demonstrated that this GMP improved gut microbiome composition, circulating markers of the tryptophan/kynurenine signaling pathway, and neuroinflammation in aged rats (43) counteracting biological processes that are involved on age-related decline of physical and cognitive function. Notably, the response of exercise training in gut microbiome and neuroinflammation is inconsistent since it seems to be attenuated in older animals, when compared with younger counterparts (14–16). Thus, we hypothesize that by adding our GMP with regular exercise training will enhance the response of exercise training in older animals.

Recently, we presented the empirical results of our GMP when combined with moderate exercise training on physical performance outcomes and on secondary healthspan indices in aged rats (29). Despite the promising findings on physical and cognitive function with this multimodal intervention, we were unable to dissect the mechanistic intricacies by which Ang (1–7) modulation, exercise training, or the interaction of all influenced physical and cognitive responses. Therefore, herein we use a multiomics approach (i.e., microbiome, targeted metabolomics, and transcriptomics) to explore the potential underlying mechanisms driven by our combined intervention on blood serum, prefrontal cortex, hippocampus, colon, liver, and skeletal muscle in aged male rats. Our hypothesis is that combining both interventions (i.e., GMP + moderate exercise training) will provide additional benefits than each intervention alone. We believe that this consecutive line of studies will provide more insight into the cellular and molecular mechanisms underpinning this multimodal strategy and analyze if the effects that this multimodal intervention exerts on host gut, brain, and muscle in late life are additive or antagonist.

## METHODS

### Experimental Design Overview

**Figure 1.**
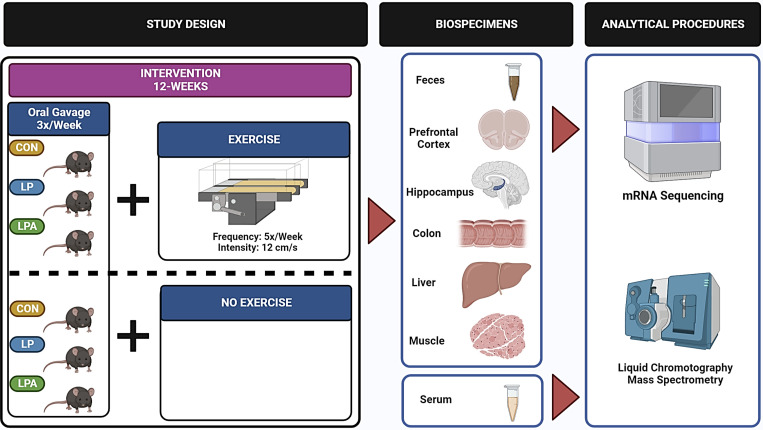
Illustrative scheme of experimental study design and analytical procedures. CON, Control; LP, *L. paracasei*; LPA, *L. paracasei* expressing Angiotensin (1–7).

This study was completed at the University of Alabama at Birmingham (UAB) and all experimental procedures were approved by the UAB Institutional Animal Care and Use Committee (IACUC-21360). The detailed experimental study design has been described previously (42, 43). Briefly, this study was a 3×2 prospective randomized trial with a total length of 12 wk. Male F344BN rats (*n* = 8–13/group) were randomized to one of three treatments: *1*) buffer (Con: *n* = 25); *2*) a probiotic-*Lactobacillus paracasei* (LP = 22); or to, *3*) a GMP-*Lactobacillus paracasei* engineered to express Ang (1–7) (LPA: *n* = 17). In addition, these groups were further randomized to a moderate exercise intervention (CONEx: *n* = 13: LPEx: *n* = 11: LPAEx: *n* = 8) or to a control group without exercise training (CONSed: *n* = 12: LPSed: *n* = 11: LPASed: *n* = 9). The treatment groups were orally gavaged with 2 × 10^11^ CFU/kg body weight of probiotics or an equal volume of buffer three times per week (Fig. 1). Groups randomized to the exercise intervention (CONEx: *n* = 13: LPEx: *n* = 11: LPAEx: *n* = 8) performed a treadmill intervention, 5 days per week for 12 wk. Prior to the exercise intervention, exercising groups underwent a 2 wk treadmill acclimatization. On the first day of exercise acclimatization, rodents were placed on the treadmill for 10 min while the machine was off. From the second to fifth day of the habituation period, the treadmill was set to 8 cm/s and then increased by 1 cm/s until it reached 12 cm/s. The total session lasted 5 min. Following acclimatization, the exercise groups performed training sessions at a speed of 12 cm/s throughout the experiment. Sedentary groups (CONSed: *n* = 12: LPSed: *n* = 11: LPASed: *n* = 9) were placed on the treadmill for 10 min/day while the machine remained off to control for any stress response from the handling and transfer of exercising animals.

### Animals

Eighty-two male F344BN rats aged 24 mo were obtained from the National Institute on Aging colony (Harlan Laboratories, Indianapolis, IN). Sixty-three survived throughout the intervention and were used in this study. The animals were individually housed at UAB under standard conditions [12-h light/12-h dark cycle (0600–1800) at 20–23°C] in a facility accredited by the American Association for Accreditation of Laboratory Animal Care. Rodents were fed a standard chow diet (18% kcal from fat, no sucrose, 3.1 kcal/g diet 2018; Harlan, Teklad, Madison, WI) and their health status, body weight, and food intake were monitored weekly. Baseline demographic characteristics of animals used in this study are described in Table 1. Female animals were not included in this study due to the unavailability of female animals at the NIH colony at the beginning of the study.

**Table 1.** Baseline demographic characteristics of male aged

Rat ID	Weight, g	Fat, g	Lean, g	Microbiome	Metabolomics	Transcriptomic				
						PFC	Hippocampus	Colon	Liver	Muscle
ConSed3	573.00	153.07	377.83	X	X	X	X	X	X	X
ConSed4	568.00	116.82	408.34	X	X	X	X	X	X	X
ConSed6	572.00	114.14	412.19	X	X	X	X	X	X	X
ConSed7	583.00	140.07	401.44	x	X	X	X	X	X	X
ConSed8	585.00	126.52	416.90	X	X	X	X	X	X	X
ConSed9	573.00	130.35	400.31	X	X					
ConSed10	583.00	140.07	401.44	X	X	X	X	X	X	X
ConSed11	585.00	126.52	416.90	X	X					
ConSed12	573.00	130.35	400.31	X	X					
ConSed13	555.00	110.56	405.16	X	X					
ConSed14	528.00	98.80	390.03	X	X					
ConSed15	557.00	112.86	401.51	X	X					
ConEx5	564.00	132.17	389.73	X	X	X	X	X	X	X
ConEx7	547.00	97.35	405.86	X	X	X	X	X	X	X
ConEx9	540.00	103.60	395.66	X	X	X	X	X	X	X
ConEx10	553.00	131.46	379.43	X	X	X	X	X	X	X
ConEx11	508.00	99.40	367.30	X	X	X	X	X	X	X
ConEx12	558.00	120.33	392.73	X	X	X	X	X	X	X
ConEx13	613.00	138.72	419.57	X	X					
ConEx14	528.00	102.22	387.59	X	X					
ConEx15	541.00	93.82	408.08	X	X					
ConEx16	575.00	137.31	395.14	X	X					
ConEx17	592.00	134.26	413.79	X	X					
ConEx18	645.00	181.08	415.11	X	X					
ConEx19	531.00	84.81	410.03	X	X					
LPSed4	555.00	111.90	403.35	X	X	X	X	X	X	X
LPSed5	548.00	103.60	399.50	X	X	X	X	X	X	X
LPSed7	514.00	94.40	379.70	X	X	X	X	X	X	X
LPSed8	516.00	93.33	384.77	X	X	X	X	X	X	X
LPSed9	556.00	99.21	411.05	X	X					
LPSed10	556.00	99.21	411.05	X	X	X	X	X	X	X
LPSed13	582.00	132.53	399.78	X	X	X	X	X	X	X
LPSed14	538.00	110.52	390.53	X	X					
LPSed16	542.00	119.67	383.99	X	X					
LPSed17	548.00	94.96	414.44	X	X					
LPSed18	588.00	129.19	416.38	X	X					
LPEx3	530.00	101.52	389.10	X	X	X	X	X		X
LPEx4	521.00	105.37	382.18	X	X	X	X	X	X	X
LPEx5	557.00	114.24	403.53	X	X					
LPEx6	498.00	82.35	378.05	X	X	X	X	X	X	X
LPEx7	538.00	106.13	394.34	X	X					
LPEx8	504.00	91.95	378.75	X	X	X	X	X	X	X
LPEx9	534.00	109.67	382.51	X	X					
LPEx10	569.00	115.62	408.32	X	X	X	X	X	X	X
LPEx11	536.00	123.91	372.75	X	X	X	X	X	X	X
LPEx12	564.00	125.16	393.83	X	X					
LPEx13	539.00	98.03	399.94	X	X					
LPASed4	555.00	86.81	392.66	X	X	X	X	X	X	X
LPASed5	532.00	99.89	395.56	X	X	X	X	X	X	X
LPASed6	481.00	82.74	366.14	X	X	X	X	X	X	X
LPASed7	522.00	98.66	386.20	X	X	X	X	X	X	X
LPASed8	565.00	101.93	419.22	X	X					
LPASed9	515.00	96.34	377.39	X	X	X	X	X	X	X
LPASed11	536.00	115.5	376.15	X	X	X	X	X	X	X
LPASed12	552.00	114.91	392.39	X	X					
LPASed13	537.00	101.76	395.24	X	X					
LPAEx4	562.00	105.21	415.97	X	X	X	X	X	X	X
LPAEx6	505.00	69.33	399.57	X	X	X	X	X	X	X
LPAEx7	522.00	105.63	380.77	X	X	X	X	X	X	X
LPAEx8	537.00	99.59	400.37	X	X					
LPAEx9	531.00	102.59	386.59	X	X	X	X	X	X	X
LPAEx11	483.00	96.10	348.57	X	X	X	X	X	X	X
LPAEx12	545.00	113.11	387.36	X	X	X	X	X	X	X
LPAEx13	609.00	136.52	423.53	X	X					

CONEx, control + exercise group; CONSed, control + sedentary group; DEG, differential expressed genes; FDR, false discovery rate; LPEx, *Lactobacillus paracasei* + exercise group; LPSed, *Lactobacillus* paracasei + sedentary group; LPAEx, *Lactobacillus paracasei* expressing angiotensin (1–7) + exercise group; LPASed, *Lactobacillus* paracasei + sedentary group.

### Euthanasia and Tissue Collection

After the intervention, rodents were euthanized via rapid decapitation and tissue was dissected immediately. Whole blood was collected, processed for serum and stored at −80°C after centrifugation. Prefrontal cortex, hippocampus, colon, liver, and tibialis anterior (TA) muscle were collected from all animals, snap-frozen in liquid nitrogen, and stored at −80°C until further analysis. One rat was excluded from euthanasia and tissue harvesting due to premature death in the morning of scheduled euthanasia. Feces were collected directly from the rodent’s colon, stored in commercially available preservative Para-Pak tubes (Meridian Bioscience Inc., Cincinnati, OH), flash frozen, and stored at −80°C until analysis.

### Outcomes

#### Fecal microbiome taxonomy, composition, and abundance.

##### Microbiome sequencing.

Fecal samples were processed for taxonomic analysis by the UAB Microbiome Core as previously described (43). Briefly, 16S-based polymerase chain reaction (PCR) assays with unique bar-coded primers were used to amplify the V4 region of the 16S rRNA to create an “amplicon library” (44). Following electrophoresis, UV illumination was used to visualize the PCR band products, which were excised and purified. Paired-end reads of ∼250 bp from the V4 region of 16S RNA were analyzed. Following quantitation with Pico Green and after de-multiplexing, Fastq was used as the raw data files format. Sequences were grouped into amplicon sequence variants (ASV) and fecal microbiota composition and abundance were analyzed. ASVs were then filtered, clustered, and summarized at different hierarchical levels (e.g., phylum, class, etc.). The taxonomic identification of the ASVs sequences was obtained using Mothur and compared with daSILVA 16S database.

##### Microbiome data analysis.

Data were imported in R (v.4.1.2) and R studio (v.2021.09.1, Boston, MA) for use with the Phyloseq package (45). Diversity within- and between samples were assessed using α and β diversity indexes, as previously described (43). Alpha diversity was calculated using Inverse Simpson and Shannon–Wiener Index values. Beta diversity was measured using the Bray Curtis Dissimilarity index, Unweighted Uni-Frac, and Weighted Uni-Frac distances. Principle coordinate analysis (PCoA) was performed to visualize the matrix dissimilarity between all samples and a PERMANOVA on both exercise and probiotic groups was performed. Analysis of compositions of microbiomes (ANCOM) with bias correction was used to test for differential abundance of individual ASVs at several levels across exercise and probiotic treatment groups using modified versions of previously published ANCOM scripts (46, 47). Briefly, raw ASV counts were filtered for any ASVs present in at least 30% of all samples. The ANCOM detection limit was set to the default value of 0.7 and was run on centered log ratio transformed (CLR) count data using a Benjamini–Hochberg corrected significance level of 0.05 and adjusted for cohort grouping as a covariant.

#### Targeted metabolomics of the serum tryptophan/kynurenine pathway.

##### Sample preparation.

Serum concentrations of kynurenine/tryptophan signaling pathway metabolites were quantified. Briefly, serum samples were aliquoted at 25 µL for extraction. All samples were spiked with 5 µL of internal standards (IS) consisting of tryptophan13C11, serotonin-d4, kynurenine-d4, kynurenic acid-d5, and picolinic acid-d4. Calibration curves in bovine serum albumin (BSA) were prepared for targeted quantitation of tryptophan (100–25,600 ng/mL), serotonin (2.5–25,600 ng/mL), kynurenine (10–640 ng/mL), kynurenic acid (1.25–1,280 ng/mL), and picolinic acid (7.5–3,840 ng/mL). Quality control (QC) samples were also prepared in BSA at concentrations within the range of the calibration curves. All calibration curves, QCs, and samples were extracted by protein precipitation using 200 µL of 8:1:1 acetonitrile:methanol:acetone with 0.1% formic acid. Supernatant from each was transferred to a clean tube after centrifugation, dried down under a gentle stream of nitrogen, and reconstituted with 25 µL of 0.1% formic acid in water for liquid chromatography-mass spectrometry (LC-MS) analysis.

##### Liquid chromatography-mass spectrometry.

Targeted LC-MS quantitation of tryptophan, serotonin, kynurenine, kynurenic acid, and picolinic acid was performed on a Thermo Q-Exactive Orbitrap mass spectrometer with Dionex UHPLC and autosampler. All samples were analyzed in positive heated electrospray ionization with a mass resolution of 35,000 at *m*/*z* 200 as separate injections. Separation was achieved on an ACE 18-PFP 100 × 2.1 mm, 2 µm column using a gradient with mobile phase A as 0.1% formic acid in water and mobile phase B as acetonitrile. The flow rate was 350 µL/min with a column temperature of 25°C and injection volume of 2 µL. Run time was 20.5 min. Peak areas of each analyte and corresponding internal standard in the calibrators, QCs, and samples were integrated using Xcalibur. A calibration curve was generated by plotting nominal concentration of the analyte in the calibrators versus peak area ratio of analyte and IS. QCs and samples were quantitated against the calibration curve.

##### Metabolomics data analysis.

Differential metabolomics analysis were completed using GraphPad Prism (v.9.2.0) using a mixed-model analysis of variance to determine probiotic + exercise interaction effects with Bonferroni correction for multiple comparisons. Main effects of exercise and probiotics were investigated when no meaningful interaction effects were observed. The statistical threshold for meaningful differences was set at a *P* value <0.05.

#### mRNA transcriptomics of the prefrontal cortex, hippocampus, colon, liver, and tibialis anterior muscle.

The mRNA sequencing data contained information of *n* = 4–6 animals/group and the baseline characteristics are described in Table 1.

##### RNA sequencing.

Tissue samples were delivered to Novogene, LLC for mRNA isolation, library preparation, and mRNA sequencing. Libraries were generated with NEBNext UltraTM RNA Library Prep Kit for Illumina (New England BioLabs) following the manufacturer’s recommendations. Briefly, mRNA was purified from total RNA using polyT, oligo-attached magnetic beads, followed by fragmentation using divalent cations in NEBNext First Strand Synthesis Reaction Buffer (5×). First-strand cDNA was synthesized using random hexamer primer and M-MuLV reverse transcriptase (Rnase H). Second strand cDNA synthesis was then performed using DNA polymerase I and Rnase H. Remaining overhangs were converted into blunt ends via exonuclease/polymerase activities. After adenylation of the 3′ ends of DNA fragments, NEBNext Adaptor with hairpin loop structure were ligated to prepare for hybridization. To select cDNA fragments of 250–300 bp, the library fragments were purified with AMPure XP system (Beckman Coulter). Then, 3 μL USER Enzyme (New England BioLabs) was used with size-selected, adaptor-ligated cDNA at 37°C for 15 min, which was followed by another incubation of 5 min at 95°C before PCR. PCR was performed with Phusion High-Fidelity DNA polymerase, Universal PCR primers and Index (X) Primer. Finally, PCR products were purified (AMPure XP system) and library quality was assessed on the Agilent Bioanalyzer 2100 system. The clustering of the index-coded samples was performed on a cBot Cluster Generation System using PE Cluster Kit cBot-HS (Illumina) according to the manufacturer’s instructions. After cluster generation, the library preparations were sequenced on an Illumina platform and 150 bp paired-end reads were generated.

##### RNAseq data processing and analysis.

Paired-end fastq files generated from sequencing were checked for quality using FastQC adapters (48) and low-quality reads (PHRED < 20) using Trim Galore and CutAdapt. Alignment to the rat genome (Rnor_5.0) and gene counts were then generated using Spliced Transcripts Alignment to a Reference (STAR). Although RNAseq analysis packages like limma (49) and DESeq2 (50) can be utilized to generate linear models to test for interaction effects among groups, complex designs with relatively small samples sizes may be served better using the simpler approach of the ∼group design in DESeq2 (49, 50). The ∼group design collapses all levels into a single factor and is similar to adding interaction terms. As our principal interest was determining whether the LPAEx group had meaningful changes in gene expression compared with the other groups, we utilized the ∼group design for exploratory analyses to investigate differentially expressed genes (DEGs) for all groups compared with LPAEx in each respective tissue group. The statistical threshold for meaningful differences was set at an false discovery rate (FDR) < 0.10 (Benjamini-Hochberg) and an absolute log2fold change (|LFC|) > 1.0. DESeq2 was completed using R (v.4.1.2) and R Studio (v.2021.09.1). Gene lists with less than 15 genes were excluded from follow-up pathway analyses, while gene lists with > 15 DEGs were input into Enrichr (51). Gene set analyses were completed using BioPlanet 2019 as the reference database. The list of all Enrichr significant inputs (i.e., FDR < 0.10) can be found in Supplemental Tables S1–S5. The R package, *UpSetR* (v.1.4) was used to generate the UpSet plots (52) for visualizing the LPAEx comparisons across tissues. An integrative network analysis was also performed on microbiome ASVs data at genus taxonomy level, on tryptophan/kynurenine metabolome and mRNA sequencing data of the prefrontal cortex and liver using partial least squares regression analysis. This multivariate approach for integrative network analysis was set to include associations with |*r*| > 0.4 and a statistical level of significance of *P* < 0.05. The multilevel community detection method was performed using xMWAS (53), a data-driven integration and network analysis tool (v.0.552, accessed using the server-hosted Shiny app: https://kuppal.shinyapps.io/xmwas/) using only the CONSed and LPAEx group samples. Finally, raw and processed data files have been uploaded to Gene Expression Omnibus. The supplementary figures and tables were uploaded to Figshare public access data repository (https://doi.org/10.6084/m9.figshare.20750677).

## RESULTS

### Fecal Microbiome Composition and Abundance

#### Diversity within samples—α diversity analysis.

**Figure 2.**
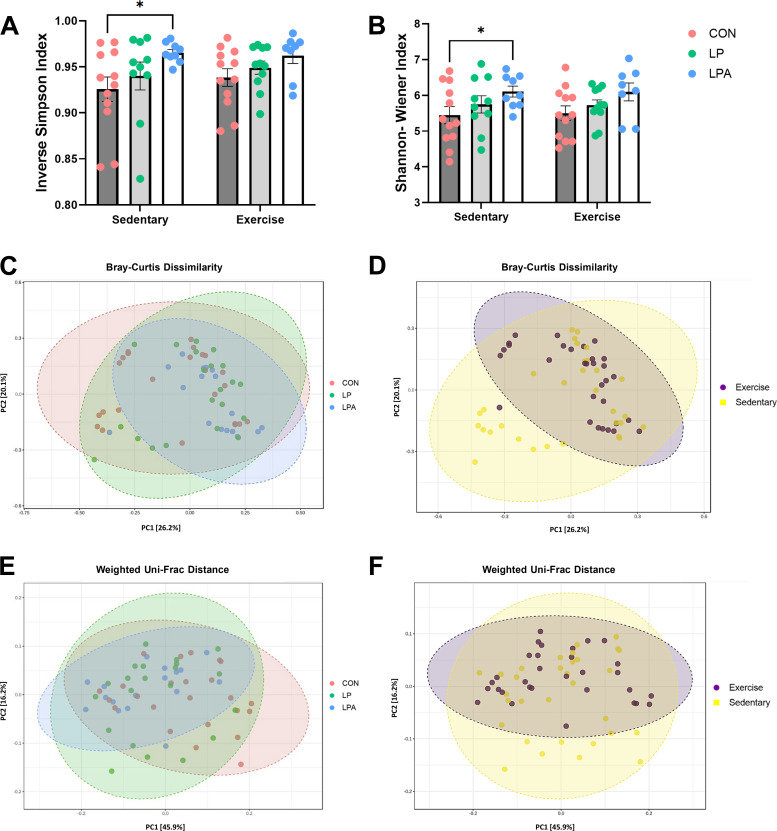
Fecal microbiome diversity analysis in aged male rats. Diversity within samples was assessed using Inverse Simpson and Shannon–Wiener indexes (*A* and *B*). Data were calculated using a mixed model analysis of variance followed by Bonferroni post hoc multiple comparisons correction. Probiotic treatment significantly altered the Inverse Simpson and Shannon–Wiener indexes showing a higher α diversity among probiotic groups. Diversity between samples was calculated using a permutational multivariable analysis of variance on Bray–Curtis dissimilarity and on Weighted Uni-Frac distances and were visually displayed through the principal coordinate analysis (PCoA) using the relative percentage abundance of amplicon sequence variants. The Bray–Curtis dissimilarity distance indicated a significant effect of exercise, but not of probiotic treatment (*C* and *D)* whereas the Weighted Uni-Frac distance revealed a main effect of probiotic but not of exercise training (*E* and *F).* Data are expressed as group means ± 1 SE; (*n* = 63 rats). *Statistical significance *P* ≤ 0.05. CON, Control; LP, *L. paracasei*; LPA, *L. paracasei* expressing Angiotensin (1–7).

Given that our treatment intervention used bacteria as a vehicle to deliver Ang (1–7) and can potentially influence gut microbiome composition, we used microbiome 16S RNA sequencing on fecal samples collected following our multimodal intervention to assess changes in the composition of gut microbiota. Inverse Simpson and Shannon–Wiener Index values were used as measures of α diversity, or the diversity within each sample. Statistical analysis revealed that there were no significant interaction effects between treatment factors (i.e., probiotic × exercise intervention) on both α diversity indices (*P* ≥ 0.05). Similarly, there was no significant main effect of exercise in rats receiving LPA, LP, and CON treatments (*P* ≥ 0.05 for all comparisons; Fig. 2, *A* and *B*). However, there was a main effect of probiotic treatment, on both indices. The Inverse Simpson (*F*[2,56] = 4.44; *P* = 0.02) and Shannon–Wiener (*F*[2,56] = 4.27; *P* = 0.02) indices showed significantly higher α diversity among male rats receiving our experimental GMP.

#### Diversity between samples—β diversity analysis.

The distance measured between each pair of samples (i.e., β-diversity) was calculated using PERMANOVA on Bray–Curtis dissimilarity, Unweighted Uni-Frac, and Weighted Uni-Frac distances. The results were visually displayed through the principal coordinate analysis (PCoA) using the relative percentage abundance of amplicon sequence variants (ASVs) across the probiotic and exercise intervention groups. On Bray–Curtis dissimilarity distance, there were neither significant interaction effects between treatment factors (*F*[2,56] = 1.13; *P* = 0.31) nor a main effect of probiotics (*F*[2,56] = 1.50; *P* = 0.09). However, exercise significantly altered β diversity across groups (*F*[1,56] = 2.22; *P* = 0.03; Fig. 2, *C* and *D*). The Unweighted Uni-Frac distance did not reveal any statistically significant interaction nor a main effect of each treatment factor (Probiotic: (*F*[2,56] = 1.42; *P* = 0.10; Exercise (*F*[2,56] = 1.23; *P* = 0.19); Supplemental Fig. S1). The Weighted Uni-Frac distance showed a main effect of probiotic treatment (*F*[2,56] = 2.66; *P* = 0.01) but not of exercise training (Fig. 2, *E* and *F*).

### Differential Composition Analysis

**Figure 3.**
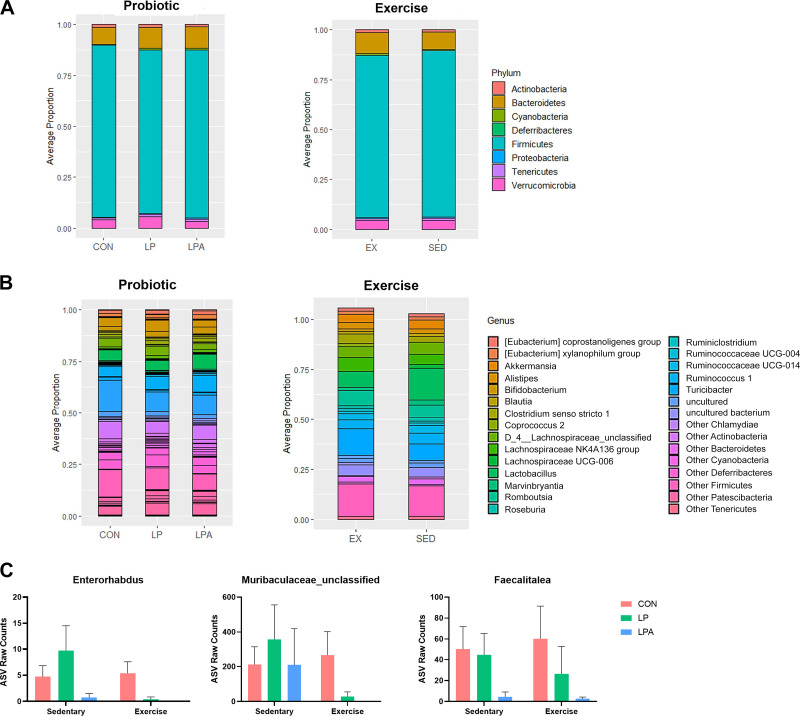
Fecal microbiome differential composition abundance in aged male rats at different taxonomic levels. Analysis of compositions of microbiomes (ANCOM) with bias correction was used to test for differential abundance of individual amplicon sequence variants (ASVs) at phylum and genus level. Raw ASV counts were filtered for any ASVs present in at least 30% of all samples. ANCOM detection limit was set to the default value of 0.7 and was run on centered log ratio transformed count data using false discovery rate (FDR) < 0.05. At the phylum level, there were no significant differences across either treatment factors (*A* and *B*). Similarly at genus level, there were no significantly different genera across exercise treatment groups but there were three genera significantly altered by probiotic treatment (*C*). Data are expressed as group means ± 1 SE; (*n* = 63 rats). CON, Control; LP, *L. paracasei*; LPA, *L. paracasei* expressing Angiotensin (1–7).

Analysis of composition of microbiomes (ANCOM) with bias correction was utilized to examine ASVs that had statistically different abundance between treatment groups across different taxonomic levels. At the phylum level (Fig. 3*A*), the relative abundance of the different communities did not significantly differ between probiotic nor exercise intervention. ANCOM analyses were repeated at the genus level (Fig. 3, *B* and *C*) and showed that three genera were significantly altered by probiotic supplementation: *Faecalitalea*, *Entororhabdus*, and *Muribaculaceae unclassified*. Exercise did not significantly change any genera.

#### Targeted metabolomics of serum tryptophan/kynurenine pathway.

**Figure 4.**
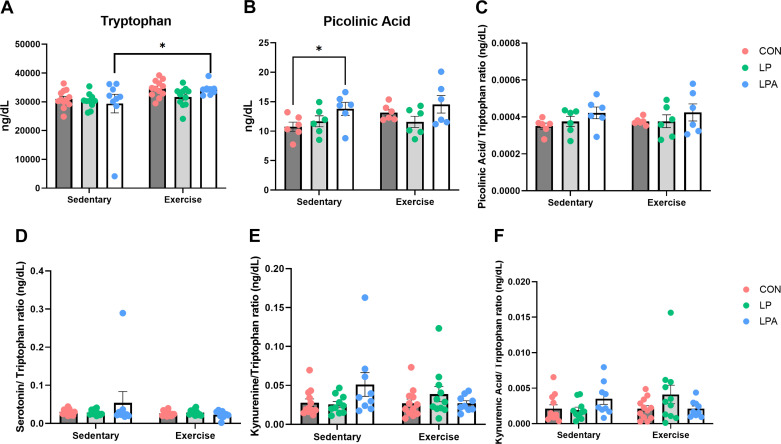
Differential metabolomics analysis on serum of tryptophan and kynurenine signaling pathway metabolites in aged male rats. Data were calculated using a mixed models’ analysis of variance with Bonferroni post hoc multiple comparisons correction. Individual tryptophan concentration on blood serum was significantly affected by exercise training (*A*) and picolinic acid concentration was significantly altered by our genetically modified probiotic (GMP) probiotic (*B*). There were no significant effects of each treatment factor across the different ratios (*C–F*). Data are expressed as group means ± 1 SE; (*n* = 63 rats). *Statistical significance *P* ≤ 0.05. CON, Control; LP, *L. paracasei*; LPA, *L. paracasei* expressing Angiotensin (1–7).

To investigate potential changes in the tryptophan/kynurenine signaling pathway that may modulate RAS components, gut microbiota, and physical and cognitive health outcomes on both treatment factors, we used LC-MS to explore systemic alterations of these metabolites. Differences between treatment factors on individual metabolite concentrations and normalized to tryptophan concentration are depicted in Fig. 4. Serum analysis showed no significant interaction effects between both treatment factors (*P* ≥ 0.05 for all comparisons). However, there was a significant main effect of exercise on tryptophan concentration (*F*[1,57] = 8.198; *P* = 0.01) and a significant effect of probiotic on picolinic acid (*F*[2,57] = 3.768; *P* = 0.04) (Fig. 4, *A* and *B*). Serum concentrations of serotonin, kynurenine, and kynurenic acid did not reveal any significant effect of exercise nor probiotic (Supplemental Fig. S2). Circulating levels of each metabolite adjusted to tryptophan concentration showed no significant main effect of each treatment factor across the different metabolite’s ratios (Fig. 4, *C–F*).

#### Multi-tissue differential gene expression, enrichment analysis, and integrative network analysis.

##### Prefrontal cortex.

**Figure 5.**
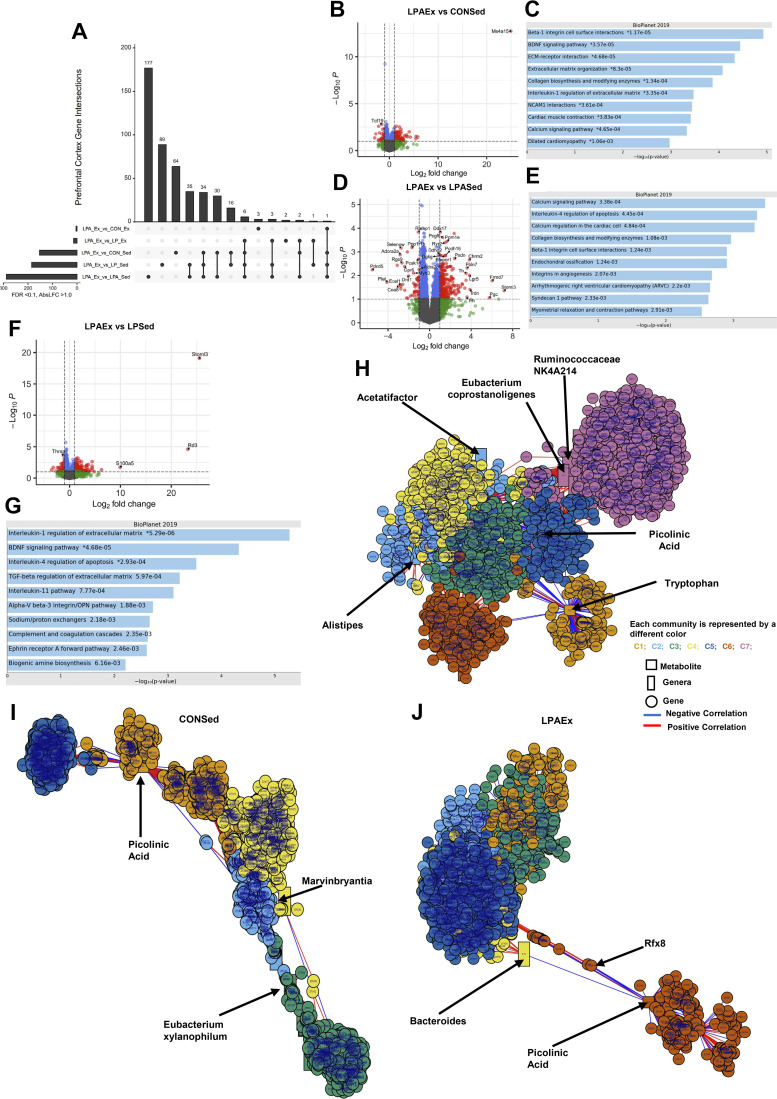
Differential gene expression analysis on prefrontal cortex of aged male rats. Differential expressed genes (DEGs) were calculated using DESeq 2 for all groups compared to *Lactobacillus paracasei* expressing angiotensin (1–7) + exercise group (LPAEx) using false discovery rate (FDR) < 0.10 and log2fold change > 1.0. Upset graph displays the transcriptomics analysis of prefrontal cortex using LPAEx as the comparator across groups (*A*). Data showed a significant effect of exercise training when compared with sedentary groups. The volcano plots display the DEGs in each comparison. Our multimodal intervention modulated 170 genes in the comparison of LPAEx against control + sedentary group (CONSed) (upregulated 140 DEGs; *B*). Exercise training altered 317 genes (upregulated 262 DEGs; *D*) in the comparison between LPAEx vs. *L. paracasei* expressing angiotensin (1–7) + sedentary group (LPASed) and 203 genes between LPAEx vs. *L. paracasei* + sedentary group (LPSed) (upregulated 163 DEGs; *F*). The enrichment pathway analysis presents the top 10 most significant pathways of the DEGs upregulated in each comparison (*C, E*, and *G*). These analyses showed an association with markers of calcium signaling, extracellular matrix re-modeling, and of brain-derived neurotrophic factor signaling pathways. Integrative network analysis of tryptophan/kynurenine pathway metabolites, 16S RNA microbiome data at genus level, and prefrontal cortex mRNA sequencing data using partial least square at |*r*| > 0.4 and *P* < 0.05 using the xMWAS data integration tool (*H*). The multilevel community detection method revealed seven communities (*C1*–*C7*) associated to tryptophan metabolite (*C1*: gold) and picolinic acid (*C5*: dark blue) along with different *genera* and genes. In addition, the multilevel community detection method using only the control sedentary male animal samples (i.e., CONSed) set at |*r*| > 0.8 and *P* < 0.05 detected five different communities (*C1*–*C5*; *I*). Using only the genetic modified probiotic expressing angiotensin (1–7) combined with exercise training samples (i.e., LPAEx) revealed six different communities (*C1*–*C6*; *J*) correlated to picolinic acid (*C6*: orange) and Bacteroides (*C4*: yellow) along with the different genera and genes. In the volcano plots, gray dots stand for nonsignificant genes; blue dots present genes that had FDR < 0.10; green dots show genes with log2fold change > 1.0; red dots represent the DEGs with both criteria FDR < 0.10 and log2fold change > 1.0; (*n* = 4–6 rats).

Unique and shared DEG profiles across all comparisons for the prefrontal cortex are shown in the UpSet plot in Fig. 5*A* and Table 2. There were 170 DEGs for the LPAEx versus CONSed comparison with 140 genes upregulated and 30 downregulated (Fig. 5*B*). *Ms4r15*, *Frmd7*, and *Itga10* were the most upregulated with *Lemd2, Dma1*, and an unannotated gene (i.e., *ENSRNOG00000058482*) the most downregulated genes. Gene set enrichment analyses highlighted extracellular matrix regulation and brain-derived neurotrophic factor (BDNF) signaling as key processes associated with the DEG signature (Fig. 5*C*). Likewise, there were 317 DEGs altered in the LPAEx versus LPASed comparison (i.e., 262 up- and 55 downregulated genes; Fig. 5*D*). The most significant genes upregulated in this comparison were *Stoml3*, *Frmd7*, and *Pgc* whereas *Pdcd5, Plat*, and an unannotated gene (i.e., *ENSRNOG00000050660*) were the most downregulated genes. The follow-up enrichment analyses revealed an association with calcium signaling pathway processes (Fig. 5*E*). Similarly, there were 203 DEGs modulated in the comparison of LPAEx against LPSed (i.e., 163 up- and 40 downregulated genes; Fig. 5*F*). The genes significantly upregulated by this comparison were *Stoml3*, *Rd3*, and *S100a5* whereas the most downregulated genes were the unannotated gene *ENSRNOG00000049394*, *Pcsk1n*, and *Lemd2*. Follow-up enrichment analysis of DEGs showed an association with interleukin 1 regulation of extracellular matrix and brain-derived neurotrophic factor (BDNF) signaling pathways (Fig. 5*G*). Comparisons to the other exercise groups exhibited minimal differences in DEGs with the LPEx upregulating 14 DEGs whereas the comparison with CONEx revealed only 4 DEGs (3 up- and 1 downregulated genes) (Table 2). The integrative network analysis revealed seven communities (|*r*| > 0.4 and *P* < 0.05) associated to the metabolome, the microbiome, and the prefrontal cortex mRNA sequencing data (Fig. 5*H*). Among the most apparent correlations, tryptophan (C1: gold) was negatively associated to different genes of *communities 5* and *6*. The genus *Akkermansia* (C6: orange) was positively correlated with kynurenine and kynurenic acid (|*r*| = 0.5; *P* < 0.05). When using only the control sedentary male animal samples (i.e., CONSed; Fig. 5*I*), the multilevel communities detection method identified five communities of tight correlated metabolites, genera, and genes while using only the LPAEx samples (Fig. 5*J*) revealed six different communities. Most of the multilevel communities pairwise correlation results showed negative associations of microbiome and metabolome on LPAEx samples, except for *Lactobacillus* genus (C1: gold). This genus was positively correlated to serotonin and kynurenic acid (|*r*| > 0.8; *P* < 0.05) whereas picolinic acid (C6: orange) was negatively correlated with *Bacteroides* genus (C4: yellow).

**Table 2.** Differentially gene expression and pathway analysis using LPAEx as the comparator group on prefrontal cortex

Group Compared with LPAEx	Total DEGs	Upregulated	Downregulated	Top Three Genes Up/Downregulated	Top Relevant BioPlanet 2019 Pathways	FDR
*LPASed*	317	262	55		***All DEGs***	
					Calcium signaling pathway	0.006
					Hypothetical network for drug addiction	0.012
				*Stoml3*	***Upregulated DEGs***	
				*Frmd7*	Calcium signaling pathway	0.072
				*Pgc*	Collagen biosynthesis and modifying enzymes	0.072
				*Pdcd5*	***Downregulated DEGs***	
				*Plat*	GPCR ligand	<0.001
				*ENSRNOG00000050660*	Neuroactive ligand-receptor interaction	0.012
*LPEx*	14	14	0		***All DEGs:***	N/A
				*C1ql2*	***Upregulated DEGs***	
				*Scara5*		N/A
				*Twist1*		
					***Downregulated DEGs***	N/A
*LPSed*	203	163	40		***All DEGs***	
					Interleukin-1 regulation of extracellular matrix	0.007
					BDNF signaling pathway	0.038
				*Stoml3*	***Upregulated DEGs***	
				*Rd3*	Interleukin-1 regulation of extracellular matrix	0.002
				*S100a5*	BDNF signaling pathway	0.010
					Interleukin-4 regulation of apoptosis	0.041
				*ENSRNOG00000049*	***Downregulated DEGs***	
				*394*		
				*Pcsk1n*		N/A
				*Lemd2*		
*CONEx*	4	3	1		***All DEGs***	N/A
				*Cd74*	***Upregulated DEGs***	N/A
				*Vim*		
				*Aebp1*		
				*Tnnc2*	***Downregulated DEGs***	N/A
*CONSed*	170	140	30		***All DEGs***	
					Beta-1 integrin cell surface interactions	0.005
					BDNF signaling pathway	0.007
				*Ms4a15*	***Upregulated DEGs***	
				*Frmd7*	Beta-1 integrin cell surface interactions	0.002
				*Itga10*	BDNF signaling pathway	0.005
				*Lemd2*	***Downregulated DEGs***	
				*RT1-DMa*		N/A
				*ENSRNOG00000058482*		

The pathway analysis were performed using Bioplanet2019 as the reference database in all comparisons with > 15 genes differently expressed. Pathways with a FDR < 0.10 were considered statistically significant. CONEx, control + exercise group; CONSed, control + sedentary group; DEG, differential expressed genes; FDR, false discovery rate; LPEx: *Lactobacillus paracasei* + exercise group; LPSed, *Lactobacillus* paracasei + sedentary group; LPAEx, *Lactobacillus paracasei* expressing angiotensin (1–7) + exercise group; LPASed, *Lactobacillus* paracasei + sedentary group; N/A, not applicable.

### Liver

**Figure 6.**
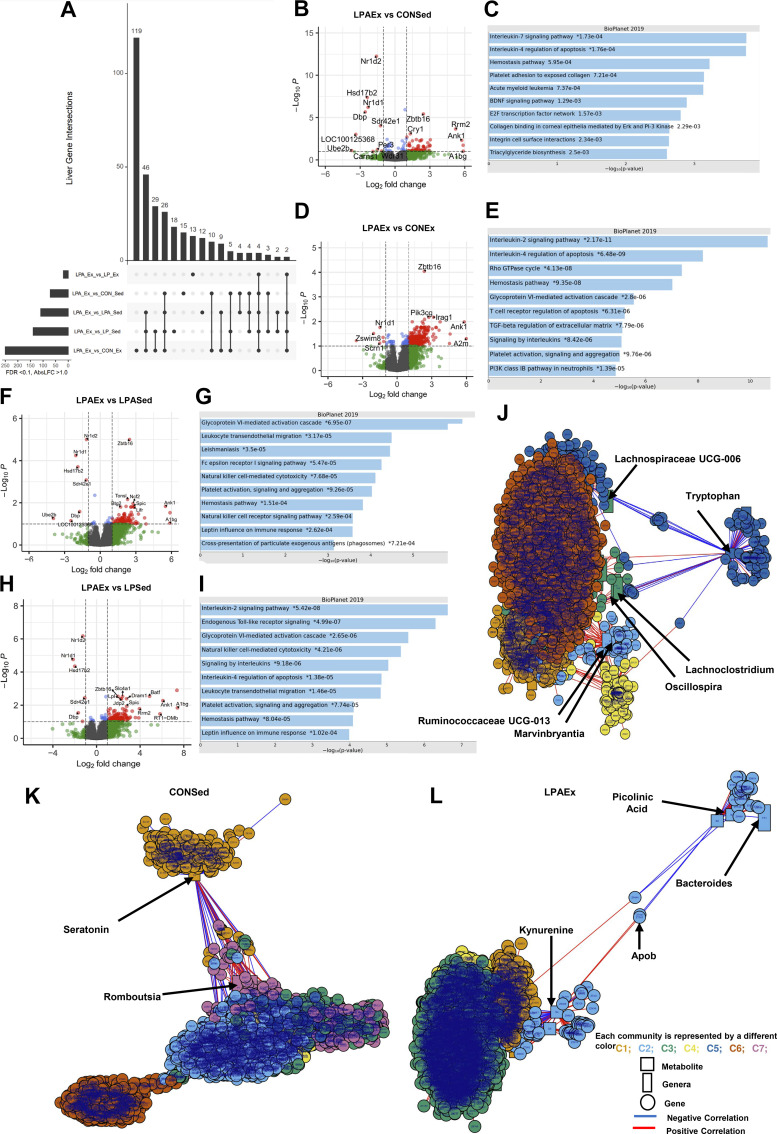
Differential gene expression analysis on liver of aged male rats. Differential expressed genes (DEGs) were calculated using DESeq 2 for all groups compared to *Lactobacillus paracasei* expressing angiotensin (1–7) + exercise group (LPAEx) using false discovery rate (FDR) < 0.10 and log2fold change > 1.0; Upset graph displays the transcriptomics analysis of liver using LPAEx as the comparator group (*A*). Our multimodal intervention [genetically modified probiotic (GMP) + exercise training) altered 73 genes [i.e., upregulated 63 in the comparison of LPAEx vs. control + sedentary group (CONSed); *B*]. In addition, DEGs analysis showed a significant effect of exercise training when compared with sedentary groups in the liver but also of probiotic treatment [i.e., upregulated 252 DEGs in the comparison between LPAEx vs. control + exercise group (CONEx); *D*]. Similarly, exercise training modulated 112 genes in the comparison between *L. paracasei* expressing angiotensin (1–7) + exercise group (LPAEx) vs. LPASed (i.e., upregulated 105 DEGs; *F*), 143 genes in the comparison between LPAEx vs. *L. paracasei* + sedentary group (LPSed) (i.e., upregulated 137 DEGs; *H*). The enrichment pathway analysis presents the top 10 most significant pathways of the DEGs upregulated in each comparison (*C, E, G*, and *I*). Follow-up pathway analysis showed that most genes upregulated in the liver were related to inflammation signaling markers. Integrative network analysis of tryptophan/kynurenine pathway metabolites, 16S RNA microbiome data at genus level and liver mRNA sequencing data using partial least square at |*r*| > 0.4 and *P* < 0.05 using the xMWAS data integration tool (*J*). The multilevel community detection method revealed seven communities (*C1*–*C7*) that included tryptophan metabolite (*C5*: dark blue) as well as the different *genera* and genes correlated. In addition, the multilevel community detection method using only the control sedentary male animal samples (i.e., CONSed) set at |*r*| > 0.8 and *P* < 0.05 detected seven different communities (*C1*–*C7*; *K*). Using only the genetic modified probiotic expressing angiotensin (1–7) combined with exercise training samples (i.e., LPAEx) revealed four communities (*C1*–*C4*; *L*) associated to picolinic acid and kynurenine metabolites (*C2*: light blue) along with the different genera's and genes. In the volcano plots, gray dots stand for nonsignificant genes; blue dots present genes that had FDR < 0.10; green dots show genes with log2fold change > 1.0; red dots represent the DEGs with both criteria FDR < 0.10 and log2fold change > 1.0; (*n* = 4–6 rats).

We observed a strong transcriptional response in the liver across comparisons, with unique and shared DEGs across comparisons shown in Fig. 6*A* and Table 3. The LPAEx versus CONSed comparison resulted in 73 DEGs (63 up- and 10 downregulated; Fig. 6*B*). The most significant genes upregulated by this comparison were *A1bg, Ank1*, and *Rrm2* whereas *Ube2b*, an unannotated gene (i.e., *ENSRNOG00000048864*) and *Dbp* were the most downregulated. The gene set enrichment pathway analyses highlighted interleukin 7 signaling pathway and interleukin 4 regulation of apoptosis (Fig. 6*C*). The comparison of LPAEx versus CONEx group exhibited the highest amount of DEGs with a total of 258 genes (252 up- and 6 downregulated; Fig. 6*D*) while the comparison versus LPEx had minimal differences in gene expression (*n* = 19 total). The most significant genes upregulated by the LPAEx versus CONEx comparison were *A2m* and *Ank1* whereas an unannotated gene (i.e., *ENSRNOG00000004419*), *Zswim8, Nfr*, *and Nr1d1* were the most downregulated genes. Gene set enrichment pathway analyses were associated with interleukin 2 signaling and interleukin 4 regulation of apoptosis (Fig. 6*E*). The other comparisons with abundant DEGs were against the sedentary groups with most of the different genes expressed being upregulated. The comparison versus the LPASed resulted in 112 DEGs (105 up- and 7 downregulated; Fig. 6*F*). The most significant genes upregulated by LPAEx against LPASed were an unannotated gene (i.e., *ENSRNOG00000037549*), *A1bg*, and *Ank1* whereas *Ube2b*, the unannotated gene *ENSRNOG00000048864* and *Nr1d1* were the most downregulated genes. The follow-up enrichment pathway analyses highlighted glycoprotein VI-mediated activation cascade and leukocyte transendothelial migration processes (Fig. 6*G*). The comparison against LPSed resulted in 143 different genes (137 up- and 10 downregulated) and upregulated *A1bg, the unannotated gene (i.e., ENSRNOG00000009329)* and *Ank1* whereas downregulated *Nr1d1*, *Hsd17b2*, and *Dbp* genes (Fig. 6*H*). The follow-up enrichment analyses showed associations with interleukin 2 signaling pathways and endogenous Toll-like receptor signaling (Fig. 6*I*). The comparison against the LPEx group resulted in 22 DEGs (11 both up- and downregulated; Table 3). The integrative network analysis on metabolome, gut microbiome at genus level and liver transcriptome data detected seven different communities (|*r*| > 0.4 and *P* < 0.05; Fig. 6*J*). Similarly to the integrative network analysis in the prefrontal cortex, the most apparent correlation in the liver was with tryptophan (*C5*: dark blue) that was negatively associated to different *genera* and genes of *community 3* (*C3*: green) including *Lachnospiraceae UCG-006*, *Lachnoclostridium*, and *Oscillospira.* When using the multilevel community’s detection method only on CONSed samples (|*r*| > 0.8 and *P* < 0.05; Fig. 6*K*), this method revealed SEVEN different communities with the most apparent correlation related to serotonin (*C1*: gold) and to different genes and *genera* of *community 7* (*C7*: purple). When using only the LPAEx samples, the multilevel community network method detected four communities tightly correlated (|*r*| > 0.8 and *P* < 0.05; Fig. 6*L*). The most apparent correlation is within *C2* (light blue). This community revealed a negative correlation of picolinic acid, to *Bacteroides* genus and *ApoB* gene, in contrast with kynurenine metabolite.

**Table 3.** Differentially gene expression and pathway analysis using LPAEx as the comparator group on liver

Group Compared with LPAEx	Total DEGs	Upregulated	Downregulated	Top Three Genes Up/Downregulated	Top Relevant BioPlanet 2019 Pathways	FDR
*LPASed*	112	105	7		***All DEGs***	
					Glycoprotein VI-mediated activation cascade	<0.001
					Leukocyte transendothelial migration	0.007
					Natural killer cell-mediated cytotoxicity	0.007
				*ENSRNOG000000*	***Upregulated DEGs***	
				*37549*	Glycoprotein VI-mediated activation cascade	0.001
				*A1bg*	Leukocyte transendothelial migration	0.010
				*Ank1*	Interleukin-4 regulation of apoptosis	0.010
				*Ube2b*	***Downregulated DEGs***	
				*ENSRNOG000000*		N/A
				*48864*		
				*Nr1d1*		
*LPEx*	19	11	8		***All DEGs***	
					Cholesterol biosynthesis	<0.001
					Lipid and lipoprotein metabolism	<0.001
				*Ank1*	***Upregulated DEGs***	N/A
				*Rrm2*		
				*Hba-a3*		
				*Abcg8*	***Downregulated DEGs***	
				*Abcg5*		N/A
				*Tlcd2*		
*LPSed*	143	137	6		***All DEGs***	
					Interleukin-2 signaling	<0.001
					Endogenous Toll-like receptor signaling	<0.001
				*A1bg*	***Upregulated DEGs***	
				*ENSRNOG000000*	Interleukin-2 signaling	<0.001
				*09329*	Endogenous Toll-like receptor signaling	<0.001
				*Ank1*		
				*Nr1d1*	***Downregulated DEGs***	
				*Hsd17b2*		
				*Dbp*		
*CONEx*	258	252	6		***All DEGs***	
					Interleukin-2 signaling	<0.001
					Interleukin regulation of apoptosis T cell receptor regulation of apoptosis	<0.001, <0.001
				*A2m*	***Upregulated DEGs***	
				*Ank1*	Interleukin-2 signaling	<0.001
				*Faahl*	Interleukin regulation of apoptosis	<0.001
					T cell receptor regulation of apoptosis	<0.001
				*ENSRNOG000000*	***Downregulated DEGs***	
				*04419*		N/A
				*Zswim8*		
				*Scrn1*		
*CONSed*	73	63	10		***All DEGs***	
					Circadian rhythm	0.023
					Interleukin-7 signaling pathway	0.028
				*A1bg*	***Upregulated DEGs***	
				*Ank1*	Interleukin-4 regulation of apoptosis	0.030
				*Rrm2*	Interleukin-7 signaling pathway	0.030
				*Ube2b*	***Downregulated DEGs***	
				*ENSRNOG00000048864*		N/A
				*Dbp*		

The pathway analysis were performed using Bioplanet2019 as the reference database in all comparisons with >15 genes differently expressed. Pathways with a FDR < 0.10 were considered statistically significant. CONEx, control + exercise group; CONSed, control + sedentary group; DEG, differential expressed genes; FDR, false discovery rate; LPEx, *Lactobacillus paracasei* + exercise group; LPSed, *Lactobacillus* paracasei + sedentary group; LPAEx, *Lactobacillus paracasei* expressing angiotensin (1–7) + exercise group; LPASed, *Lactobacillus* paracasei + sedentary group; N/A, not applicable.

### TA Skeletal Muscle, Hippocampus, and Colon

**Figure 7.**
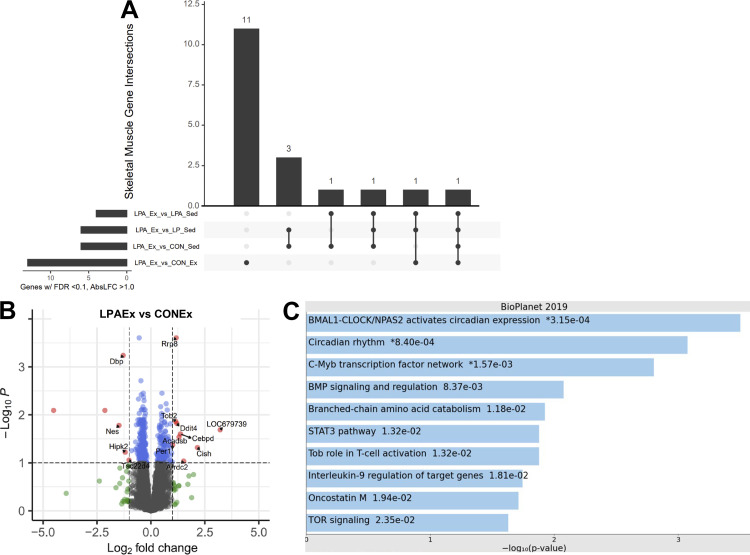
Differential gene expression analysis on tibial anterior skeletal muscle of aged male rats. Differential expressed genes (DEGs) were calculated using DESeq 2 for all groups compared with *Lactobacillus paracasei* expressing angiotensin (1–7) + exercise group (LPAEx) using false discovery rate (FDR) < 0.10 and log2fold change > 1.0. Upset graph displays the transcriptomics responses on skeletal muscle using LPAEx as the comparator across groups (*A*). Data showed a small effect of our probiotic treatment. Probiotic treatment altered 15 DEGs in the LPAEx vs. control + exercise group (CONEx) comparison (9 up- and 6 downregulated; *B*). The enrichment pathway analysis presents the top 10 most significant pathways of the DEGs upregulated in this comparison (*C*). Follow-up pathway analysis showed that most genes upregulated in the skeletal muscle were associated with circadian rhythm signaling pathways. In the volcano plots, gray dots stand for nonsignificant genes; blue dots present genes that had FDR < 0.10; green dots show genes with log2fold change > 1.0; red dots represent the DEGs with both criteria FDR < 0.10 and log2fold change > 1.0; (*n* = 4–6 rats).

There were only 2–14 DEGs across almost all LPAEx comparisons in the TA skeletal muscle, hippocampus, and colon, thus, there were no follow-up pathway analyses performed. The exception to this were 15 DEGs in the LPAEx versus CONEx comparison in the TA skeletal muscle and these DEGs were enriched for BMAL1-CLOCK/NPAS circadian rhythm expression (Fig. 7, *A–C*). The most significant genes upregulated by this comparison were the unannotated gene *ENSRNOG00000023387, Cish*, and Arrdc2 whereas two unannotated genes (i.e., *ENSRNOG00000019736* and *ENSRNOG00000022637*) were the most downregulated. In addition, LPAEx comparison versus CONSed in the hippocampus resulted in 19 DEGs (5 up- and 14 down regulated) but the pathway enrichment analysis did not exhibit statistical relevance (FDR > 0.10). Further summarization of these data is provided in Supplemental Fig. S3 and Supplemental Tables S1–S3.

## DISCUSSION

Ang (1–7) is a promising therapeutic target for modulating RAS functions in multiple tissues relevant to age-related diseases (41, 54), but current pharmacodynamics properties limit its applicability in clinical care. Herein, we present the findings from multiomics and multi-tissue profiling following an intervention combining administration of GMP-expressing Ang (1–7) with exercise training in an aging male rat model. Our multimodal strategy modulated the composition of fecal microbiota within- and between groups after the 12-wk intervention and altered the transcriptional responses in the prefrontal cortex and liver, with minimal effects on the hippocampus, colon, and TA skeletal muscle. These findings support, at least partially, our main hypothesis that our GMP expressing Ang (1–7) has a beneficial additive effect when combined with regular exercise training on gut microbiome, tryptophan metabolome, and on transcriptomics responses in the prefrontal cortex and liver in aged male rats.

Specifically, our GMP enhanced α diversity in both the Simpson and Shannon–Wiener indexes and also revealed a statistically significant main effect of probiotic treatment on the weighted Uni-Frac distance, a β-diversity metric that accounts for both the presence/absence of shared taxa between samples as well as their abundance. In contrast, the Bray–Curtis dissimilarity distance showed a significant main effect of exercise training but not of probiotic treatment. These discrepancies are related to how both distance matrices are calculated to assess β-diversity across groups since the Bray–Curtis dissimilarity matrix only considers abundance. Conversely, the weighted Uni-Frac along with the taxa’s abundance and presence/absence of ASVs between samples also uses the phylogenetic tree to assess close related taxa’s as more similar and thus, it provides a more robust β-diversity information of microbial communities between groups. At the phylum level, we did not observe any significant change between treatments nor exercise factors. Despite the benefits of exercise training on gut microbiome composition and function, similar rodent and human studies have shown also mixed findings with exercise training on gut microbiome at different taxonomic levels (9, 11, 13, 55). We are unsure as to the reasons for this finding, but distinct training protocols (voluntary wheel vs. forced treadmill running) have shown different gut microbiome compositions in rodents (56). We will look to replicate findings as well as explore exercise effects with distinct training protocols both in female and male rats. Still, at the genus level, our GMP expressing Ang (1–7) decreased the relative abundance of three genera—the *Enterorhabdus*, *Muribaculaceae unclassified*, and *Faecalitalea*. Notably, the genus *Enterorhabdus* belongs to *Actinobacteria* phylum that assembles several families of Gram-positive bacteria. In several preclinical studies of obesity and type 2 diabetes, higher relative abundance of *Enterorhabdus* was positively correlated with body weight, weight gain percentage, and fat mass accumulation as well as with blood lipids, glucose, and insulin resistance (57–62). Furthermore, changes of *Enterorhabdus* genera were associated with the pathophysiological development of Alzheimer’s disease (63). In contrast, preclinical studies using dietary interventions (58, 63) and a gut-target approach with a *L. paracasei* strain (i.e., K56) intervention (57) reduced the relative abundance of this genera in obese mice and in rats with Alzheimer’s disease dysbiosis. Likewise, *Faecalitalea*, a genera from *Firmicutes* phylum, has been proposed as a gut-derived marker to monitor patients with adenoma, which is associated with an increased risk of colorectal cancer (64). In a case-controlled study, patients with advanced adenomas had higher abundance of *Faecalitalea* and were positively correlated with specific adenoma amino acids (64). Consistent with these dietary and gut-derived approaches, our multimodal treatment strategy (GMP + exercise training) reduced the relative abundance of the *Enterorhabdus* and *Faecalitalea* genera suggesting that our combined treatment modulates gut microbiota composition by altering the abundance of endotoxic genera. In addition, the results of our integrative network analysis further support the distinct associations of several genera with metabolome and gene expression in the prefrontal cortex and liver. For instance, the relative abundance of the genus *Bacteroides* was negatively correlated with picolinic acid concentration in the prefrontal cortex and liver in the LPAEx group whereas the relative abundance of the genus *Akkermansia* was positively correlated with kynurenine and kynurenic acid on prefrontal cortex, which is consistent with our previous work (43).

One empirical question that lingered from prior work was the potential mechanistic link between our GMP and the gut-brain axis. This bidirectional network axis is distinctly modulated by gut microbiota (65–68) and by ACE2 (69, 70), the enzyme that converts Ang I or II to Ang (1–7), and influences the concentration of tryptophan directly or indirectly through downstream metabolites (71, 72). Therefore, we hypothesized that our combined treatment modulated this bidirectional network, altering the circulating levels of tryptophan and kynurenine metabolites. Notably, after the 12-wk multimodal intervention, we only observed a muted systemic response on tryptophan and picolinic acid concentrations with our targeted metabolomics approach that were differently altered by exercise and our GMP. We did not find any other statistically significant effect on serotonin, kynurenine, and kynurenic acid upregulated by our multimodal intervention. However, our multilevel community network analysis did show distinct communities of tryptophan/kynurenine pathway metabolites in the prefrontal cortex and in the liver correlated to different genera and genes suggesting that tryptophan metabolism has a key role between the gut and other peripheral organs like the brain and liver. In an interesting study of chronic restraint stress measuring metabolic changes in tryptophan metabolites in the prefrontal cortex, hippocampus, serum, and in different gut sections, Deng et al. (73) observed a similar null response in circulating levels of tryptophan metabolites, except on kynurenine. Notably, the metabolic response after the chronic restrain stress intervention in tryptophan metabolites was more pronounced locally on the hippocampus, small intestine, and colon, in contrast with the null effect seen on serum, suggesting a specific “local” effect on the central nervous system and on the gut. Based on this evidence and in the results of our multilevel community network analysis, we wonder if our multimodal intervention upregulated a similar “local” tissue response that we could not capture with our analytical approach. Of note, mean elevations of picolinic acid (although not statistically significant) by our GMP expressing Ang (1–7) are consistent with our previous work (43) and were supported also by the results of our integrative network analysis that showed distinct correlations with genes and the genus *Bacteroide* in the prefrontal cortex and liver in our animal model. Picolinic acid has been proposed as a neuroprotective agent (74) and this may indicate that at least partly, our GMP may affect neurodegeneration through changes in systemic metabolites of tryptophan metabolism—though further follow up is needed to confirm this mechanistic link between our GMP and the gut-brain axis.

Finally, we explored the underlying transcriptome of multiple tissues to identify molecular signatures modulated by our multimodal intervention. We hypothesized that our multimodal intervention upregulates important physiological pathways involved in anti-inflammation [evidence from prior studies (43)] on the gut, brain, and muscle that may influence cellular transcriptional responses in peripheral organs. The largest effect on the transcriptome in the prefrontal cortex across all LPAEx comparisons was mostly driven by the exercise response after the 12-wk intervention. For example, exercise training upregulated 262 DEGs in the LPAEx versus LPASed comparison. This altered gene expression was associated with calcium signaling and collagen biosynthesis signaling pathways, which further supports the role of our intervention in neuronal activity plasticity and remodeling, which when deregulated, can lead to neurodegeneration via complex mechanisms involved in cell death (75). In the brain, calcium is a key signaling ion, essential to control synaptic activity and memory, a process that leads to the activation of specific calcium-dependent signal transduction pathways and implicates key protein effectors (75). In turn, collagen serves as a regulator of cell differentiation and is required for preserving peripheral nerve myelination, function, and structure (76). Nonetheless, in the prefrontal cortex, our combined intervention (i.e., LPAEx vs. CONSed comparison) resulted in the upregulation of 140 genes and was associated with β-1 integrin cell surface interaction and brain-derived neurotrophic factor (BDNF) pathways. These signaling pathways are involved in relevant biological functions associated with the regulation of neurogenesis and cell remodeling. In fact, integrins are transmembrane cell adhesion molecules that regulate cellular growth, proliferation, migration, signaling, and cytokine activation along with apoptosis and tissue repair (77). Similarly, BDNF is a neurotrophic factor involved in plastic changes related to learning and memory. It regulates both excitatory and inhibitory synaptic transmission and activity-dependent plasticity (78). Taken together, the prefrontal cortex transcriptomic changes suggest that our multimodal intervention modulates cellular mechanisms associated with exercise that support neurogenesis, neuro-remodeling, synaptic plasticity, and neural activity that collectively are crucial physiological mechanisms for cognitive and memory maintenance.

Interestingly, the liver was the only tissue that had a sufficient number of DEGs against the LPEx group, suggesting a potential Ang (1–7) probiotic effect, with the DEG profile enriched for cholesterol metabolism. The exercise effect was similarly observed in the liver, however the largest unique gene set across liver comparisons was observed in the LPAEx versus CONEx group (119 unique DEGs), also suggesting a potential probiotic effect. The vast majority of gene set enrichment analyses of the liver across LPAEx comparisons, including all three sedentary groups, were associated with inflammatory signaling pathways. For example, most of the comparisons were enriched for leukocyte and interleukin signaling including interleukin-2 (IL-2) signaling, IL-4, and T cell regulation of apoptosis, and leukocyte trans-endothelial migration. IL-2 is a pleiotropic cytokine that controls the differentiation and homeostasis of both pro- and anti-inflammatory T-cells, and influence various lymphocyte subsets during differentiation, immune response, and homeostasis (79, 80). IL-4 is also a pleiotropic cytokine with an important role in regulating antibody production, hematopoiesis, and inflammation, and the development of effector T cell responses (81, 82). Similarly, IL-7, a cytokine known for its growth-promoting effects on T-lymphocytes, B-lymphocytes, and natural killer cells (83) mediates both the innate and acquired immune system. Collectively, these findings suggest that our multimodal intervention significantly affects the anti-inflammatory cytokine transcriptional response in the liver stimulating markers of the innate and acquired immune systems cells namely on cell-growth, differentiation, and homeostasis. However, further research is needed to confirm this anti-inflammatory effect and the exact physiological mechanism driving this modulation. For instance, is this effect mediated by: *1*) the enhancements on local and systemic Ang (1–7) concentration (69, 70); *2*) or through exercise improved intestinal immune function that reduces systemic gut-associated endotoxemia (84); *3*) or due to gut-liver axis that mediates the interaction of tryptophan metabolites with immune system receptors (84); 4) or in fact, an interaction of all these factors.

Surprisingly, the transcriptional response on skeletal muscle was modest, with almost no effect of exercise. In fact, after the 12-wk intervention, the comparison with the most DEGs was LPAEx versus CONEx, suggesting the potential for a peripheral effect of our probiotic intervention. This differential gene expression signature was associated with the circadian rhythm. Little is known about exercise as a zeitgeber to maximize health benefits but increasing evidence shows that exercise and skeletal muscle modulates a strong circadian clock. However, the complex interactions between the microbiome, exercise, skeletal muscle, and circadian rhythm are largely undetermined. In mammals, circadian clocks regulate physiological processes with day-night light cycles through a network of transcription factors that upregulates rhythmic gene expression (85) and dysregulation of this molecular clock can lead to deleterious metabolic consequences (86). Thus, further research is needed to confirm this mechanistic link.

Although we found promising findings with our multimodal intervention, our study is not without caveats. First, the small sample size and complex design in the transcriptomic analysis may have limited the statistical power. Exploratory comparisons using just the LPAEx group allowed us to find potential DEGs among all groups, but this limits complete understanding of our data set. In addition, we used thresholds for both FDRs and fold-change understanding that gene expression changes may be biologically meaningful even if they don’t meet relevant fold-changes. Second, we only used male rats in this study due to the unavailability of female animal numbers at the NIH colony, and thus, further research is needed to explore potential distinct cellular transcriptional and gut-derived cellular responses in both sexes. Third, we used a targeted metabolomics approach to assess circulating concentrations of tryptophan and kynurenine signaling metabolites due to the relevance of our previous work, but untargeted metabolomics of different tissue compartments likely would have provided additional insight. Challenges in obtaining assay materials due to supply chain issues prohibited us from measures associated with Ang(1–7)-related analytes in this particular study although we have previously published results on probiotic effects (42). Finally, the present study did not include the measurement of indoles markers, thus limiting inference on the potential clinical impact of indoles on physical and cognitive function that have been associated with neuroprotective effects (71). Still, we used gold-standard techniques in all procedures of the study design (i.e., randomization), sample collection, processing and analysis along with statistical approaches to limit the effects of confounding factors and diminish the overextrapolation of the results.

In summary, our findings suggest that our GMP has a beneficial additive effect to regular exercise training in aged male rats and may serve as an adjunct therapeutic strategy to enhance exercise training responses on gut microbiome and on prefrontal cortex and liver transcriptome in older animals. Our GMP enhanced gut microbial diversity and at least partly, mean levels of tryptophan metabolites while moderate exercise training contributed to altering the transcription responses in relevant neuro-remodeling and inflammation signaling pathways, with the largest effects seen in the prefrontal cortex and liver. However, further research is needed to determine the exact mechanisms by which the gut microbiota exerts its modulatory effects, particularly on tryptophan and kynurenine local metabolites (i.e., skeletal muscle, liver, and central nervous system) along with genes that were upregulated by our pathway analysis to confirm these findings and translate it to humans.

## DATA AVAILABILITY

The data described in the manuscript and the analytic code will be made available upon request pending approval by the corresponding author. The multi-tissue RNA sequencing data analyzed in this paper has been deposited in Gene Expression Omnibus at https://www.ncbi.nlm.nih.gov/geo/query/acc.cgi?acc=GSE213649.

## SUPPLEMENTAL DATA

10.6084/m9.figshare.20750677 Supplemental Figs. S1–S3 and Tables S1–S5: https://doi.org/10.6084/m9.figshare.20750677.

## GRANTS

This project was financially supported by the National Institute of Health/National Institute of Aging Grant R01AG054538 (to C.S.C. and T.W.B.). The work was also supported by the UAB Nathan Shock Center under Grant No. P30AG500886, Microbiome Resource (supported by NIH Grants P30CA0131148). L.C.B. is supported by a grant from the Portuguese Foundation for Science and Technology under Grant No. FCT/UIDP/00617/2020 and by the Laboratory for Integrative and Translational Research in Population Health Grant LA/P/0064/2020. E.L.Z. is supported by a T32 postdoctoral fellowship from the National Heart, Lung, and Blood Institute of the National Institute of Health under Grant No. T32HL007457. Z.A.G. is supported by a Department of Veterans Affairs Office of Research and Development RR&D Service CDA-2 Grant 1IK2RX002781. A.R.H. is supported by a T32 postdoctoral fellowship from the Eunice Kennedy Shriver National Institute of Child Health and Human Development of the National Institutes of Health Grant T32HD071866.

## DISCLOSURES

No conflicts of interest, financial or otherwise, are declared by the authors.

## AUTHOR CONTRIBUTIONS

C.S.C. and T.W.B. conceived and designed research; Y.S., Y.Y., A.B., A.V., and Q.L. performed experiments; L.C.B., Z.A.G., and A.R.H. analyzed data; L.C.B., Z.A.G., A.R.H., C.S.C., and T.W.B. interpreted results of experiments; L.C.B. and A.R.H. prepared figures; L.C.B., E.L.Z., Z.A.G., and A.R.H. drafted manuscript; L.C.B., E.L.Z., Z.A.G., A.R.H., T.B., Y.S., Y.Y., A.V., Q.L., C.S.C., and T.W.B. edited and revised manuscript; L.C.B., E.L.Z., Z.A.G., A.R.H., T.B., Y.S., Y.Y., A.V., Q.L., C.S.C., and T.W.B. approved final version of manuscript.
